# Preventive Strategies and Biomarkers in Male Reproductive Health from Multidisciplinary Perspective: Narrative Review

**DOI:** 10.3390/medicina62030566

**Published:** 2026-03-18

**Authors:** Gulnara Ispossunova, Dejan Nikolic, Mirzakarim Alchinbayev, Ardak Nurbakyt, Akmaral Aitmanbetova, Marta Bizic, Milan Lackovic, Filip Milanovic, Aiym Amangeldi, Anel Ispossunova, Jovana Kuzmanovic Pficer

**Affiliations:** 1 Department of Public Health, Asfendiyarov Kazakh National Medical University, Almaty 050012, Kazakhstan; gulnara.ispossunova@gmail.com (G.I.); a.arshabaevna@mail.ru (A.A.); 2 Department of Urology, Asfendiyarov Kazakh National Medical University, Almaty 050012, Kazakhstan; alchinbaev.m@kaznmu.kz; 3 Department of Physical Medicine and Rehabilitation, University Children’s Hospital, 11000 Belgrade, Serbia; denikol27@gmail.com; 4 Faculty of Medicine, University of Belgrade, 11000 Belgrade, Serbia; martabizic@gmail.com (M.B.); filipmilanovic333@gmail.com (F.M.); 5 Division of Extended Studies, University of California San Diego, San Diego, CA 92037, USA; 6 Department of Pediatric Urology, University Children’s Hospital, 11000 Belgrade, Serbia; 7 Harris Birthright Research Centre for Fetal Medicine, King’s College Hospital, London SE5 8BB, UK; lackovic011@gmail.com; 8 Department of Pediatric Surgery, University Children’s Hospital, 11000 Belgrade, Serbia; 9 School of General Medicine-2 Asfendiyarov Kazakh National Medical University, Almaty 050012, Kazakhstan; aiymamangeldi103@gmail.com; 10 Medical Center “DNA”, Kostanay 110000, Kazakhstan; doc.anelya91@gmail.com; 11 School of Dental Medicine, University of Belgrade, 11000 Belgrade, Serbia

**Keywords:** male reproductive health, prevention, diagnostics, treatment, biomarkers, challenges

## Abstract

The objective of this review is to synthesize current knowledge and evidence on male reproductive health by addressing preventive medicine strategies and biomarkers, as well as to provide clinicians, researchers, and policy makers with a coherent framework for prevention of male reproductive health. In this review male (in)fertility and determinants of male reproductive health as well as preventive strategies with special attention to primary, secondary and tertiary prevention in male reproductive health will be analyzed. From primary preventive measures: education, weight management, physical activity, sleep, healthy diet, alcohol and smoking consumption will be assessed, while from secondary preventive measures: sexually transmitted infection (STI) from the point of transmission prevention, testicular self-examination, hormonal testing and management, chronic diseases and semen analysis will be discussed; and from tertiary preventive measures: treatment of STI, treatment of congenital abnormalities, infertility treatment and urogenital cancer treatment will be elaborated. Additionally, biomarkers in male reproductive health will be synthesized and discussed. Bridging the gap between evidence and practice will ultimately lead to better understanding of the complex state of male reproductive health, thus minimizing the potential missed windows of opportunities in timely adequate preventive interventions implementations, as well as on time diagnostic and optimal treatment options.

## 1. Introduction

Reproductive health refers to a state of complete physical, mental, and social well-being in all matters in relation to the reproductive system, its functions and processes, and not merely the absence of diseases or infirmity [1]. It implies that people are able to have satisfying and safe sexual life, along with the capability to reproduce and the freedom to decide if, when, and how frequently to do so [1,2]. The interest for the male reproductive health surged by the emerging evidence of poor male reproductive health and somatic disorders associations, global decline in sperm count as well as the impact of paternal comorbidities on the next generation [3]. The importance of male reproductive health is in the fact that it is crucial for the men’s health and wellness [4]. Previous studies suggested that infertility affects 8–12% of couples worldwide, where male factors was reported to be between 40–50% [5,6]. In a review of literature by Kumar et al., it was stated that infertility rates are markedly higher in less industrialized nations, with a infectious diseases as one of contributors to the great proportion of infertility [5]. Moreover, it was argued that numerous health conditions can affect male infertility, as well as the fact that causes of male subfertility vary highly [6].

The rationale for this review is to provide a comprehensive and up-to-date overview of male reproductive health determinants, preventive approach and biomarkers. The objective of this review is to synthesize current knowledge and evidence on male reproductive health by addressing preventive medicine strategies and biomarkers. Moreover, this review aims to provide clinicians, researchers, and policy makers with a coherent framework for prevention of male reproductive health.

Despite the fact that this review analyze prevention strategies and biomarkers during the reproductive years, male reproductive health should be evaluated across the life course, including prepuberty, puberty, early adulthood, and later adulthood. It was previously argued that man may retain certain reproductive potential even in older age, and that advanced paternal age was negatively associated with a sperm quality as well as an increased risk of natural reproductive failure through infertility and miscarriage [7]. Moreover, paternal ageing was argued to have impact on offspring health [8].

## 2. Material and Methods

This narrative review was conducted in accordance with the SANRA (Scale for the Assessment of Narrative Review Articles) guidelines. The literature search was utilizing the PubMed and Google Scholar databases, as well as grey literature sources. No formal time restrictions were imposed, and older relevant publications were included when deemed significant to the topic. Only articles published in English were considered. The search was selective rather than systematic; studies were chosen based on their relevance to key aspects of male reproductive and sexual health, as determined by the authors’ expertise and professional judgement. Titles and abstracts were screened by the authors to identify pertinent studies.

The search terms employed encompassed reproductive health, male, men, sperm, semen, fertility, infertility, sexual function, sexual dysfunction, sexual health, sexually transmitted diseases, sexually transmitted infection, testicular cancer, testicular tumor, gynecomastia, male breast cancer, chronic diseases, chronic medical conditions, hypospadias, epispadias, congenital abnormalities, posterior urethral valve, congenital genitourinary anomalies, genitourinary tumors, genitourinary malignancies, bladder cancer, prostate cancer, high-risk groups, seminal plasma, sperm quality and biomarkers.

The inclusion criteria comprised systematic reviews, meta-analyses, review articles, randomized controlled trials, original research articles, guidelines, and grey literature, including statements from the World Health Organization and the United Nations. When available, higher levels of evidence, such as systematic reviews, meta-analyses, and international guidelines, were prioritized.

## 3. Male (In)Fertility and Determinants of Male Reproductive Health

In a comprehensive overview by Cavalhas-Almeida et al., it was reported that male infertility is often multifactorial, including both congenital and acquired factors that affect male reproductive system [9]. In addition to this, authors further stated that in around half of man with infertility the cause is undetermined, and this population is divided into the group with idiopathic male infertility (30–40%, characterized by abnormal seminal analysis) and unexplained male infertility (6–37%, characterized with normal seminal analysis) [9].

Regarding the male fertility, in the study of Kiess et al., it was stated that such condition is influenced and affected by several factors including: endocrinological, environmental, nutritional and sociocultural as well as secular trends [10]. Furthermore, in the review by Chen et al., it was suggested that there is an association between male reproduction and genetic, infectious as well as chronic comorbid conditions and that the diagnosis of male fertility is associated with disease risk in the future such as metabolic disease, cancer and mortality [11]. In the systematic review by Service et al., authors pointed out that obesity, diabetes and metabolic syndrome have adverse effects on numerous parameters of male fertility from semen quality to sperm deoxyribonucleic acid integrity [12]. Additional lifestyle factors such as poor diet, physical inactivity, alcohol consumption, smoking and endocrine disruptors (certain chemicals) can contribute to obesity and reduced fertility [13]. Moreover, aging in males was shown to have negative effects on various semen analysis parameters including decrease in semen volume, sperm motility, normal morphology of the sperm and increase in DNA fragmentation [7,14]. Advanced paternal age was shown to be associated with the health problems in the offspring such as: skeletal dysplasia, psychiatric morbidity and academic morbidity [15]. Additionally, certain comorbid conditions in aging man may affect fertility like prostate cancer, hypertension, hypogonadism, depression, sexual dysfunction, etc. [16] Moreover, cryptorchidism, hypospadias, testicular tumors, varicocele and Y chromosome microdeletion can lead to azoospermia or oligozoospermia [17]. In males with prostate cancer, radical prostatectomy, pelvic radiotherapy, chemotherapy and androgen-deprivation therapy often cause irreversible infertility [18]. Furthermore, it was suggested that there is an association between hypertension and impaired semen quality in males [19]. In the study of Zhang et al., it was noticed that presence of depression was negatively associated with semen quality parameters particularly in those with shorter sleep duration [20]. Sleep deprivation was shown to be associated with decreased seminal quality, fecundability and sexual dysfunction as well as increased anti-sperm antibody levels [9]. In another study, it was argued that sleep deprivation have an influence on hypothalamic-pituitary-gonadal (HPG) axis and blood-testis barrier disruption, cortisol levels elevation and oxidative stress levels increase, impairing hormonal homeostasis and sperm quality [21].

These findings clearly demonstrate the complex and multidimensional aspects of male reproductive health as well as male infertility, implying to the necessity of multidisciplinary approach in diagnosis, prevention and treatment of affected individuals. Better understanding the core concepts of individual impacts of certain risk factors as well as their interaction in male reproductive function affection will lead to proposal of adequate, evidence-based and personalized interventions for optimal preventive strategies and treatment models for the males reproductive health improvement.

## 4. Prevention Strategies

Preventive strategies for the male reproductive health should be concentrated towards integration of reproductive goals with general health promotion. In line with this, modifiable risk factors of male reproductive health represent critical targets for planning and implementing prevention interventions, with a focus on education of men, weight management, physical activity promotion, smoking cessation, alcohol intake reduction, anabolic steroids avoidance, and increased awareness for occupational health measures. In Table 1, prevention levels and domains are presented.

### 4.1. Primary Prevention

Primary prevention in male reproductive health has a principal role in preventing or reducing the exposure to the modifiable risk factors, thus preserving the reproductive potential.

In a qualitative study by Poljski et al., authors suggested the need for community education of men with general information regarding sexual and reproductive health issues [22]. They further argued that there is a lack of information for general practitioners (GP) regarding certain areas of male sexual and reproductive health, thus the education of GPs is recommended as well [22]. In the review by Hall, it was stated that men should be educated and encouraged to attend GPs as well to engage in health prevention at a community level [23]. There are numerous global male health initiatives aimed to health promotion, risk assessment and reduction, community education, disease prevention, improvement of men’s health at different stages of life, health equality promotion between different groups of men, etc [24].

Weight management was shown to be among risk factors that can be modified. In a meta-analytic study by Santi et al., it was pointed out that weight loss elevates testosterone levels as well as improve the qualitative and quantitative sperm characteristics [25]. Additionally, weight loss was shown to reduce sperm DNA fragmentation index, while moderate weight loss through dietary modification in obese man could be sufficient to enhance sperm motility [26].

Regarding physical activity, it was previously reported that men who are physically active have larger proportion of motile spermatozoa versus those practicing sedentary lifestyle, and that men who practice moderate exercise can slow down age-related inflammatory processes as well as DNA damage in sperm [27]. However, in another study it was noticed that sixteen weeks of intensive cycling training in males 17–26 years of age, resulted in seminal reactive oxygen species (ROS) and malondialdehyde (MDA) increase, remaining high even after 30 days of recovery, while seminal superoxide dismutase (SOD), catalase, and total antioxidant capacity (CAT) decreased, remaining low even after 30 days of recover, implying to the fact that such level of exercise might have negative consequences on spermatozoa [28]. Moreover, in the study by Maleki et al., it was shown that seminal oxidants and antioxidants differed between elite athletes, recreationally active and non-active men, where healthier semen production was seem to be in recreationally active men [28]. These findings point to the importance of multidisciplinary approach in proposing and implementing physical activity programs in males, where special attention should be focused on the level of intensity of such interventions and whether the participants are professional or recreational.

The importance of sleep refers in the fact that quality and quantity of sleep may have an influence on male fertility, particularly semen parameters [29]. Moreover, it was stated that there is bidirectional relationship between sleep and health, and that numerous health conditions are associated with poor sleep and short sleep duration [30]. These reports clearly indicate the complex interconnection between sleep, its characteristics and health of individuals. Therefore, strategies for the increased awareness of adequate and timely sleep importance are vital as well as interventions for achieving these goals in order to maintain optimal male reproductive health and prevent its deterioration.

Healthy diet was shown to be associated with better sperm quality, implying to the possibility that nutritional interventions might have important role in male fertility preservation [31]. Furthermore, adequate antioxidants intake may improve selected semen parameters in some contexts, but evidence for live birth remains uncertain, thus routine use is debated [32,33], and food containing antioxidants was argued to have favorable effects on conventional semen parameters and sperm function improvement [34].

The importance of alcohol consumption and smoking on male fertility were previously reported. The adverse effects mechanisms of certain ingredients of cigarettes are complex regarding sperm parameters including reproductive hormones secretion [35]. Furthermore, long-term and excessive alcohol intake have an adverse effect on testicles and can induce azoospermia [35]. In a narrative review by Finelli et al., it was noticed that alcohol consumption can have numerous negative effects on male reproductive health including reduced sperm concentration, increase abnormal sperm morphology and sperm DNA fragmentation, aberrant gene methylation in sperm DNA, altered expression of RNA involved in sperm function as well as transgenerational effects (congenital heart defects, cancer, increased psychopathological disorders, etc., in offspring) [36]. Moreover, authors stated that daily alcohol consumption have negative effects on sperm volume and sperm morphology, as well as the fact that heavy drinkers have worse semen parameters then moderate drinkers [36].

### 4.2. Secondary Prevention

Secondary prevention in male reproductive health has an important role in progression or irreversible damage prevention by proposing and implementing an adequate and optimal diagnostic and early treatment interventions.

**Figure 1:**
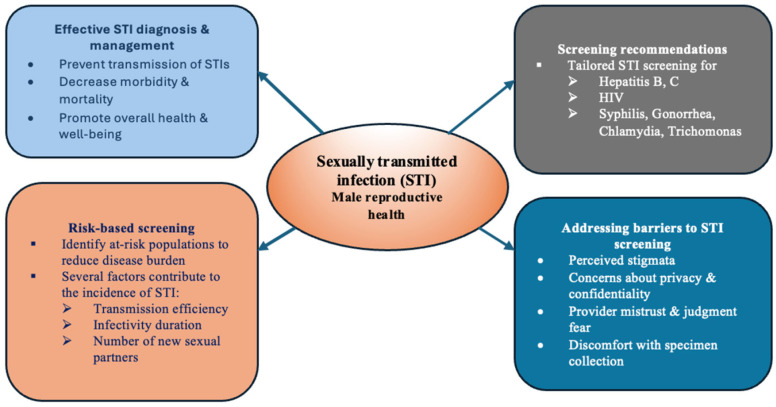
Sexually transmitted infection screening in males.

Regarding sexually transmitted infections (STI), appropriate diagnosis and treatment is vital in transmission prevention as well as decrease of the death related to the one, improving the individual’s health, sexual health and well-being [37]. One of important aspects to consider is the fact that many patients with STI are asymptomatic, thus the most cost-effective measure to reduce the burden of disease is targeted screening of populations that are at risk [38]. Several factors contribute to the incidence of STI such as transmission efficiency, duration of infectivity, and the number of new partners for infected individual per unit of time, therefore prevention or reduction of STI could be achieved by implementation of the measures including vaccination of susceptible population, reducing transmission efficacy and infectivity duration as well as change in sexual behaviors [39]. It was argued that sexual history is considered to be important first step in STI screening as well as treatment [40]. Therefore, secondary prevention of STI`s should rely primarily on risk-based screening and early treatment, rather than universal testing. In this context, targeted screening commonly includes human immunodeficiency virus (HIV), syphilis, hepatitis B and C, gonorrhea, *Chlamydia trachomatis*, and *Trichomonas*, depending on population risk [38]. In contrast, hepatitis A prevention can be achieved through vaccination with a safe and effective vaccines that have been available for decades [41]. Furthermore, it was stated that improved treatment services for STI lead to reduction in HIV incidence in an emerging HIV epidemic environment with a poor such treatment services and high STI prevalence [42]. Addressing the barriers for screening is also important. In a qualitative study from Ghana further barriers for men seeking clinical-based screening for STI’s were noticed including lack of privacy from health care providers, mistrust of healthcare providers, stigmatization fear and for those with a positive status the burden of handling thoughts [43]. In another study conducted on the population of both genders, perceived barriers to care were lack of knowledge of STI’s, lack of available services, shame connected with seeking services, cost, discrimination, long clinical waiting times as well as collection methods of urethral specimen [44]. In Figure 1, sexually transmitted infection screening in males is presented.

Due to the fact that testicular cancer is considered significant threat to male’s health between 15–34 years of age [45], there is a need for rising awareness of testicular cancer symptoms and importance of prompt clinical evaluation. In the study by Cook, it was stated that 3% of males practice regularly testicular self-examination (TSE), and that most of those that are at risk, are not aware of the existence and symptoms of the testicular cancer [45]. Moreover, in the study of Chong et al., authors argued that TSE can be useful in early detection of testicular cancer, therefore potentially improving treatment outcomes and prognosis [46]. Furthermore, in a systematic review by Saab et al., authors stated that those not performing the TSE usually were not informed about such practice, as well that majority of men perceived education for testicular cancer as a positive step for raising awareness regarding this malignancy [47]. Moreover, despite the fact that regular screening for testicular cancer is controversial, authors stated that young males should be encouraged to seek medical attention in occasions where scrotal abnormalities were discovered [47].

**Figure 2:**
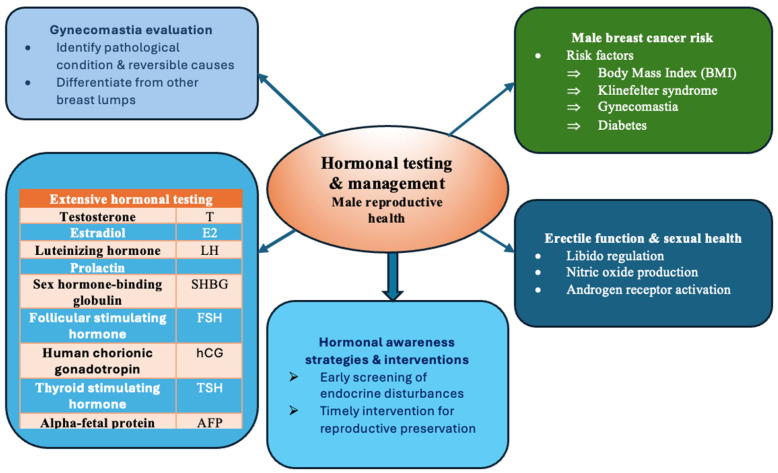
Hormonal testing and management aspects in male reproductive health.

Hormonal testing and management is also important aspect toward male reproductive health. Gynecomastia is considered frequent condition with a prevalence between 32–65% depending on age and criteria used for definition [48]. Therefore, the purpose of the assessment of gynecomastia is for underlying pathological conditions and reversible causes detection as well as discrimination from other lumps in breast area [48]. With regards to the male breast cancer, it was stated that recent rather than distant body mass index (BMI) was strongest predictor, while other conditions were also associated with such a pathology including: Klinefelter syndrome, gynecomastia and diabetes [49]. Wide plethora of hormonal testing was also suggested in gynecomastia including testosterone (T), estradiol (E2), luteinizing hormone (LH), prolactin, sex hormone-binding globulin (SHBG), follicular stimulating hormone (FSH), human chorionic gonadotropin (hCG), thyroid stimulating hormone (TSH), as well as alpha-fetal protein (AFP) [48]. Regarding erectile function and sexual health, total and free testosterone are essential, where libido regulation and nitric oxide production in penile tissues is helped by total testosterone, while free testosterone has a role in maintaining the erectile function by direct activation of androgen receptors in the corpora cavernosa [50]. Regulation of steroidogenesis and spermatogenesis is done via LH and FSH [51]. The numerous hormones, as well as their actions on different tissues and functions in males indicate how complex is the male reproductive functioning and its influence of the health in total. Therefore, strategies and interventions that will increase the awareness of these hormones and their roles in multidimensional aspects of male reproductive health are vital in early screening and treatment of diseases and conditions associated with inadequate functioning of the one. The optimal adoption of such interventions and their implementation in clinical practice both for physicians and patients will have positive effects in early recognition of even early stages of pathological processes and disbalances leading to prevention of further deterioration of male reproductive health as well as overall health and maintaining well-being. In Figure 2, hormonal testing and management aspects in male reproductive health are presented.

In the review of Sanctis et al., it was pointed out that well recognized conditions among adolescents and young male adults with present chronic diseases are pubertal growth failure, sexual development absence or delay, sexual dysfunction and infertility due to hypogonadism and defective spermatogenesis, where the causes which are multifactorial might be due to disease, associated complications or drugs [52]. Duration of diseases is also important factor to consider, since in the umbrella review by Leemans et al., it was argued that males with diabetes longer than 5 years have a higher risk for erectile dysfunction development by 3.2 times versus males with diabetes less than 5 years in duration [53]. Moreover, in a post-stroke period, male patients report erectile dysfunction [54]. Basson et al., in their study reported that numerous factors can directly or indirectly contribute to the sexual dysfunction in chronic illnesses including disruption of genital response that can be from disease, surgery, radiation, chemotherapy, treatment, pain as well as change in sexual desire from disease, reduced energy, depressed mood, impaired mobility, etc. [55] These observations clearly indicate the multidimensional influences of chronic conditions on male reproductive health and the need for raising awareness in patients, healthcare providers and community through different programs, interventions and strategies. Moreover, the importance of multidisciplinary and interdisciplinary participation in preventive, early diagnostic and early treatment interventions is vital for achieving overall optimal and best preventive outcomes by reducing functional deteriorations, complications and present disease progression. Therefore, the need for treatment and continuous monitoring of the chronic disease in males is just one aspect of the prevention strategies, while additional strategy should include measures and interventions for raising awareness of the complications that can arise affecting male reproductive health and encouraging males to take active roles in prevention as well as early detection of early-onset signs and symptoms with prompt referrals to the healthcare professional for further observations.

Semen analysis is important in male reproductive health. It is fundamental in the evaluation of male infertility and spermatogenesis [56]. Semen microbiome is important regarding male reproductive system and fertility, since its imbalance can be associated with anatomical changes in the genital tract, local inflammation, as well as alteration in sperm structure and function [57]. In a systematic review and meta-analysis of Farahani et al., it was noticed that Lactobacillus appears to protect sperm quality, while bacteriospermia had negative impact on sperm concentration as well as progressive motility and DNA fragmentation index [58]. In a U.S. cohort, men with male-factor infertility were approximately 2.8 times more likely to develop testicular cancer than those without male-factor infertility (hazard ratio, 2.8; 95% confidence interval, 1.3–6.0) [59].

In a systematic review of Finelli et al., it was suggested that computer-aided sperm analyzers (CASA) are valid alternative for semen parameters evaluation in clinical practice, however, further technological improvement of CASA are needed before they could replace human operator one day completely [60]. Moreover, even though artificial intelligence and machine learning contributed to the improvements of diagnostic efficiency, accuracy and objectivity withing advanced reproductive technologies, particularly in male fertility evaluation, there are challenges that should be further explored including lack of standardized, open-access datasets; ethical, regulatory and data security issues; further clinical validation necessity; training of healthcare professionals, etc. [61].

### 4.3. Tertiary Prevention

Tertiary prevention in male reproductive health is multidimensional, complex and multidisciplinary model that needs to be focused on minimizing long-term consequences along with optimizing psychosocial well-being and quality of life in affected young males with reproductive pathology. The goals of strategies and interventions withing tertiary prevention model should be towards the complication’s prevention and treatment.

Treatment of STI is important not just for the prevention of further disease dissemination, but for the reduction of possible complications associated with the infection on urogenital tract of the young males and other systems and organs. Further conditions and complications were described: penile discharge, penile itch, testicular pain, dysuria, urethral strictures, epididymitis as well as nonspecific one including fever, headache, fatigue, myalgia, lymphadenopathy [62,63]. Furthermore, affection of other organs and systems such as skin, kidneys, eyes, liver, rectum, oropharynx and gastrointestinal, musculoskeletal and neurological systems [62,63] were described. This clearly indicates the need for multidisciplinary and interdisciplinary approach in treatment of these patients for optimal therapeutic outcomes. Personalized interventions, educational programs and continuous monitoring along with follow-up should be suggested in treatments for these patients.

Considering congenital abnormalities of male genital system, further conditions such as cryptorchidism, hypospadias, epispadias, micropenis and posterior urethral valve (PUV) were reported [64,65,66,67]. The importance of timely recognition and treatment is due to the fact that major congenital genitourinary tract anomalies may lead to sexual and reproductive function disturbances [68].

Regarding urogenital cancers, they can affect prostate, kidney, bladder, ureter and testis [69,70], and are reported to account for 23% of all malignancies and 7% of cancer deaths [69]. The positive impact of interdisciplinary collaboration in disease management was previously recognized between urology, medical oncology, radiology, genitourinary pathology, radiotherapy, epidemiology and biostatistics [69]. For patients with prostate cancer multiple treatment options persist [71,72] as well as for bladder cancer [73]. Same applies for testicular cancer, where advancements in treatment lead to increase in survival [74], thus continuous monitoring of these patients and treatment approaches focusing on health and quality of life improvements are needed. The complex and multidimensional aspects of tumors in young males imply to the necessity for multidisciplinary and interdisciplinary approaches in timely diagnostics of potential complications, prevention of their further deteriorations as well as diseases management itself.

**Figure 3:**
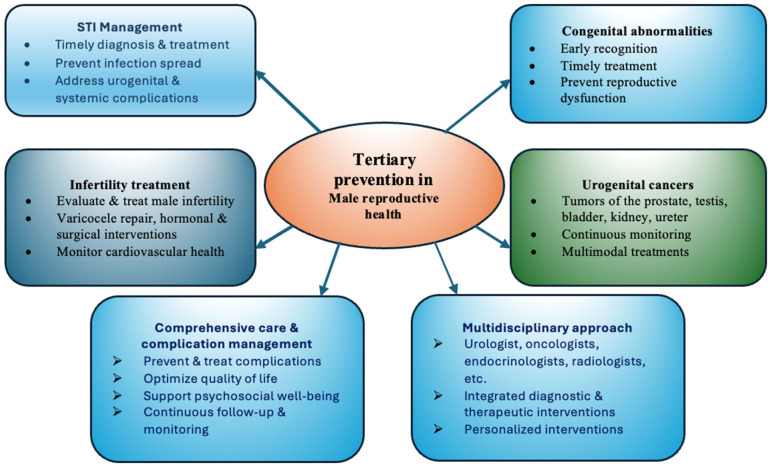
Tertiary prevention aspects in male reproductive health.

The importance of timely diagnostics and treatment of male infertility is due to the fact that male fertility diagnosis is associated with future disease risk such as metabolic disease [11], cancer [], ischemic heart disease [75] and mortality []. In male patients with infertility, specific interventions are proposed including varicocele repair, microsurgical reconstruction of obstructive conditions, correction of identifiable hormonal abnormalities and surgical relief of ejaculatory duct obstruction [76]. In Figure 3, tertiary prevention aspects in male reproductive health are presented.

## 5. Biomarkers Overview

A biomarker can be described as: “A defined characteristic that is measured as an indicator of normal biological processes, pathogenic processes, or biological responses to an exposure or intervention, including therapeutic interventions” [77]. Biomarkers represent measurable molecular, histological, radiographic, or physiological characteristics [77]. Furthermore, with regards to their putative applications, numerous subtypes of biomarkers are being defined, but it should be noticed that single biomarker might meet multiple criteria for different uses [78]. Therefore, biomarkers can be further classified into: diagnostic, monitoring, pharmacodynamic/response, predictive, prognostic, safety, multicomponent, surrogate endpoints and susceptibility/risk [77,78].

In terms of complexity, biomarkers can be described as: traditional biomarkers which are well embedded in clinical practice and research, but are generally limited in analytical complexity [79], network or modular biomarkers that can provide more quantifiable and stable approach for characterization of biomedical phenotypes or diseases then individual molecular biomarkers [80], and dynamic network biomarkers which present the method based on a model-free concept, that are capable of detecting critical states just before the bifurcation point from the normal to the disease state [80].

In order to clarify the scope of the biomarkers framework in this narrative review, the proposed synthesis is intended as a conceptual and selective framework, with an aim to illustrate how major biomarker domains can be interpreted within a preventive medicine perspective (primary, secondary and tertiary prevention). Moreover, the present framework in this review prioritizes biomarker domains that illustrate molecular, biochemical and exposure-related domains of male reproductive health.

## 6. Biomarkers and Male Reproductive Health

Even though the interest in reproductive health is increasing, the lack of biomarkers which are able to predict male fertility with high sensitivity and accuracy still lacks [81]. In the review by Parvin et al., it was stated that genomics, proteomic and metabolomic biomarkers in male infertility evaluation provide promising pathway for targeted diagnostics [82]. In a systematic review by Llavanera et al., it was argued that certain Omics including DNA structure and integrity, transcriptomics, proteomics, genomics and epigenomics as well as metabolomics might be relevant molecular biomarkers that could help in identification of infertility etiologies and fertilization prognosis [81]. It should be stressed as well that cell-free DNA, ribonucleic acid (RNA) and proteins that are found in testicles and epididymis are detected in concentrated amounts in semen, while they are absent or barely detected in blood serum [83]. Therefore, this could be potential barrier in evaluation of potential biomarkers from blood serum. Furthermore, Kovac et al., in their study pointed out that ideal biomarker should identify disease in early stage, be accurate, easily detectable and cost-effective with minimal side effects, thus discovery of such biomarkers would help avoid the necessity for invasive testing in infertile man [84].

Considering biomarkers in urogenital tumors in males, the example is prostate specific antigen (PSA), however its inability to differentiate cancer from urinary tract infection or prostatitis is known, thus turning the quest for ideal biomarker more challenging [84]. Further obstacles in the search of ideal biomarkers are in the fact that for certain biomarkers cognate protein levels do not correlate with transcript levels [85]. Moreover, despite the fact that numerous genes are expressed at higher levels in malignant tissues in comparison with the benign one, virtually no proteins or transcript were identified as uniquely elevated in cancer [85]. Also, potential biomarkers from nucleus and cytoplasm are not accessible to clinical assays [85]. In the review by Fujita and Nonomura it was stated that urine could be the promising source for the novel biomarkers development for prostate cancer [86]. In line with this, from the study by Mytsyk et al., authors revealed that PCA3 score from urine had moderate sensitivity and good specificity to differentiate clinically significant prostate cancer (PC) from non-PC [87]. Regarding testicular cancer, conventional biomarkers such as alpha-fetoprotein (AFP), lactate dehydrogenase (LDH) and beta-human chorionic gonadotropin (β-hCG) have restricted sensitivity and specificity [88]. Due to the fact that these markers have low sensitivity and specificity, novel biomarkers with improved accuracy and performance characteristics are the focus of research investigations [89]. In line with this, certain emerging biomarkers such as microRNA (miRNA), circulating tumor DNA (ctDNA) as well as circulating tumor cells (CTCs) were shown to have potential for improvement of testicular cancer management [89]. Regarding miRNA, it was stated that combination of miR-371 and miR-375 was shown to have potential in enhancing diagnostic precision in testicular cancers [88]. Furthermore, it was argued that for patients with testicular germ cell tumors (TGCT), miRNA might be reliable tool for accurate diagnosis and disease monitoring [90], however circulating miR-371a-3p was reported to be expressed in undifferentiated TGCTs, but not in teratomas [88]. Additionally, in a systematic review by Leao et al., it was revealed that levels of miR-371a-3p correlate with primary tumor mass, clinical stage, and International Germ Cell Cancer Collaborative Group risk groups [91]. Moreover, it was stated as well that high sensitivity ctDNA in testicular and renal cell carcinoma could provide valuable prognostic information [92].

Furthermore, one of important components of the male reproductive biomarker landscape is seminal plasma. It is a mixture of accessory sex glands (prostate, seminal vesicles and bulbourethral gland), epididymis, vasa deferentia and seminiferous tubule lumen secretions [93]. The importance of seminal plasma is in the fact that it regulates mechanisms of sperm capacitation and aids in the protection and maturation of sperm [93]. Semen plasma was reported to comprise of lipids, glycans, small molecule metabolites, inorganic ions, and biopolymers including cell-free DNA, RNA, micro RNAs, oligosaccharides, peptides and proteins [94]. The importance of seminal plasma evaluation is in the fact that it can serve as a potential reservoir of biomarkers that are relevant to fertility, genitourinary malignancies as well as infections [95]. In line with this, in seminal plasma, zinc was argued to be a marker of prostatic secretion, PSA as a marker of prostatic secretory capacity, fructose as a marker for assessment of seminal vesicular function, as well as other markers of prostatic activity including citric acid, γ-glutamyl transpeptidase, and acid phosphatase [95].

For characterization of inorganic constituents such as metal ions and metal-protein complexes in semen, semen plasma metallomics is used [95]. This is of particular importance since it was reported that exposure to heavy metals have adverse effect on male reproductive function [96]. Metals induce production of reactive oxygens species affecting male fertility [97]. Exposure to lead in humans was shown to correlate with decreased sperm concentration as well as reduced motility and DNA fragmentation increasement [96]. On animal models, cadmium was shown to affect semen quality parameters, testicular function and hormonal imbalance [98]. Moreover, aluminum was also reported to have adverse effects on male reproductive health including toxic effects on sperm number, morphology, viability and motility [99]. Exposure effects to arsenic on human reproduction include erectile dysfunction, testicular disorders and prostate cancer [100]. Furthermore, mercury exposure in humans can lead to reduction in sperm count, motility and viability as well as increase of abnormal sperm morphology, sperm DNA damage and sperm nuclear chromatin condensation [101]. On the other side, the selenium was reported to have important function in testosterone biosynthesis and creation and usual growth of spermatozoa [102]. Moreover, selenium control redox state, protects DNA genomic instability and mutations [102]. In the systematic review and meta-analysis of Zhao et al., the importance of zinc in male infertility was described, stating that zinc might have a role in sperm functional properties, as well as regulatory role in the capacitation and acrosome reaction processes [103]. Additionally, in seminal plasma zinc has a role in maintaining sperm chromatin stability [103].

Biomarkers derived from seminal plasma can be conceptually grouped into three domains such as glandular functional biomarkers (zinc, PSA, fructose, citric acid, γ-glutamyl transpeptidase, and acid phosphatase) [95], exposure-related biomarkers (heavy metals, metallomics) [95,96,97,98,99,100,101,102,103] and fertility-related pathophysiological biomarkers (cell-free DNA, RNA and semen proteins) [83].

Based on the reviewed literature [81,82,83,84,85,86,87,88,89,90,91,92,93,94,95,96,97,98,99,100,101,102,103], we propose a conceptual synthesis that links major biomarker categories in male reproductive health with the corresponding levels of preventions (primary, secondary, and tertiary) (Table 2).

**Figure 4:**
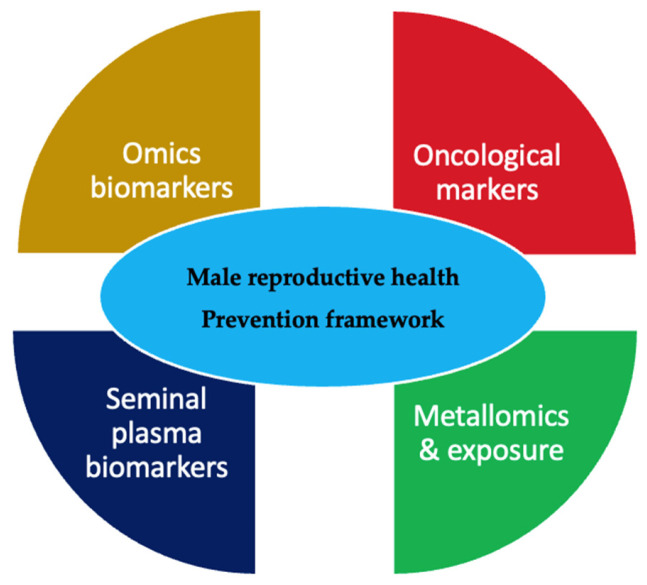
Conceptual overview of selected and emerging biomarker domains in male reproductive health.

Conceptual overview of selected and emerging biomarker domains in male reproductive health is presented in Figure 4.

Considering the different life stages of men, interpretation of different biomarkers such as semen parameters, molecular markers, imaging findings as well as endocrine profiles to male reproductive health might vary. The effects of aging of males on testicular morphology, prostate gland, semen parameters, endocrine system, reactive oxygen species, DNA mutations as well as fertilization capacity were previously described [14].

## 7. Conclusions and Future Implications

Future research on male reproductive health should focus on investigations aiming at relationships between risk factors in early life and reproductive health outcomes in later age. Moreover, preventive interventions and measures should be further explored in order to increase additional evidence-based knowledge in male reproductive health, which will lead to an improvement of reproductive, sexual and overall male health. Furthermore, male-specific reproductive health policies should be promoted across all levels of healthcare.

From the clinical point of view, development and implementation of specific reproductive risk assessment tools as well as novel biomarkers as a future diagnostic and care models in male reproductive health should be considered. Furthermore, there is a need for the development of screening and diagnostic algorithms which will have impact on early identification of sensitive groups of individuals that are at an increased risk for male reproductive health deterioration. These strategies will lead to a timely implementation of preventive and early diagnostic and treatment interventions.

Regarding future implications at the population level, there is a need for specific public-health strategies and interventions that will encourage males to be actively engaged in improving male reproductive health literacy and active participation in implementation of preventive measures.

Finally bridging the gap between evidence and practice will ultimately lead to a better understanding of the complex state of male reproductive health, thus minimizing the potential of missed windows of opportunities in timely adequate preventive interventions implementations, as well as on time diagnostic and optimal treatment options.

## Figures and Tables

**Table 1:** Prevention levels and domains.

Primary Prevention	Secondary Prevention	Tertiary Prevention
• Education • Weight management • Physical Activity • Sleep • Healthy diet • Alcohol risk reduction • (avoid heavy/binge drinking; counsel moderation) • Smoking cessation	• Diagnosis and treatment of sexually transmitted infections (STI) for transmission prevention • Testicular self-examination (TSE) • Hormonal testing and management • Education, diagnosis and treatment of chronic diseases • Semen analysis	• Treatment of sexually transmitted infections (STI) • Treatment of congenital abnormalities • Urogenital cancer treatment • Infertility treatment

**Table 2:** Author-proposed conceptual synthesis linking biomarker categories in male reproductive health with levels of prevention based on the reviewed literature.

Biomarker Category	Specific Biomarkers	Level of Prevention
Omics-based biomarkers	Genomics, transcriptomics, proteomics, metabolomics, epigenomics	Secondary prevention
Cell-free nucleic acids and proteins	Cell-free DNA, RNA, seminal proteins	Secondary prevention
Seminal plasma biochemical markers	Zinc, fructose, citric acid, γ-glutamyl transpeptidase, acid phosphatase, PSA	Secondary prevention
Metallomics biomarkers	Zinc, selenium	Primary prevention
Heavy metal exposure indicators	Lead, cadmium, mercury, arsenic, aluminum	Primary prevention
Prostate cancer biomarkers	PSA, PCA3 (urinary biomarker)	Secondary prevention
Testicular cancer classical biomarkers	Alpha-fetoprotein (AFP), β-hCG, lactate dehydrogenase (LDH)	Secondary and tertiary prevention
Emerging oncological biomarkers	microRNA (miR-371a-3p, miR-375), circulating tumor DNA (ctDNA), circulating tumor cells (CTCs)	Secondary and tertiary prevention

Author’s proposed conceptual synthesis based on references [81,82,83,84,85,86,87,88,89,90,91,92,93,94,95,96,97,98,99,100,101,102,103].

## Data Availability

Not applicable.
